# Spatiotemporal patterns of urban heat in indoor and outdoor microclimates

**DOI:** 10.1088/1748-9326/ae09bc

**Published:** 2025-10-28

**Authors:** Yusuf Jamal, Courtney C Murdock, Rajendra Kumar Baharia, Rajesh Sharma, Keshav Vaishnav, Vikas Desai, Vijay Kohli, Ajeet Kumar Mohanty, Mercedes Pascual, Sachin Sharma, Anup Anvikar, Michael C Wimberly

**Affiliations:** 1 Department of Geography and Environmental Sustainability, University of Oklahoma, Norman, OK, United States of America; 2 Department of Entomology, Cornell University, Ithaca, NY, United States of America; 3 Indian Council of Medical Research, National Institute of Malaria Research, New Delhi, India; 4 Health Department, Ahmedabad Municipal Corporation, Ahmedabad, India; 5 Vector-Borne Diseases Department, Surat Municipal Corporation, Surat, India; 6 Urban Health and Climate Resilience Centre of Excellence, Vesu Urban Health Center, Surat, India; 7 New York University, New York, NY, United States of America; 8 Data Institute for Societal Challenges, University of Oklahoma, Norman, OK, United States of America

**Keywords:** microclimate, wet bulb temperature, heat stress, urban heat island effect, indoor–outdoor, land cover, building density

## Abstract

As global temperatures rise due to climate change, urban heat islands have emerged as an important public health concern, significantly exacerbating heat stress in urban populations. Meteorological data is critical for assessing heat stress, and localized microclimate data provide more precise measurements of heat hazards than traditional weather station data. Our study explored microclimate patterns in space and time in tropical cities with rapidly growing urban populations and warming climates. We established a microclimate monitoring network with sensors measuring air temperature and relative humidity throughout two large cities in Gujarat, India. We collected hourly microclimate data on temperature and humidity from April 2023 to May 2024 from paired indoor/outdoor sensors at 48 homes in Ahmedabad and 45 homes in Surat. We summarized dry bulb (*T*) and wet-bulb (*T*_w_) temperatures at indoor and outdoor locations, compared temporal patterns across seasons and times of the day, and investigated relationships with urban land cover. Indoor and outdoor microclimates had different diurnal variations, with distinctive patterns during the monsoon compared to other seasons. Building volume had warming effects and vegetation had cooling effects on minimum *T* and *T*_w_, particularly at outdoor locations. In contrast, building volume had cooling effects and vegetation had warming effects on maximum *T* and *T*_w_, particularly at indoor locations. Temperatures were consistently cooler at locations with higher albedo, and relationships with water were weaker and more variable. A model comparison found significant differences in land cover effects for indoor versus outdoor locations. Given the increasing occurrence of heat waves and climate-related health threats in western India and other tropical areas, it will be essential to account for the different spatial and temporal patterns of indoor and outdoor microclimates to more precisely identify locations and timings of temperature extremes.

## 1. Introduction

Global temperatures are rising due to climate change, directly and indirectly affecting human health worldwide through various pathways (McMichael *et al* 2006). In addition to temperature, short-term and long-term variations of other climatological variables like humidity, wind speed, precipitation, and solar radiation have been reported to affect human health (Kinney 2008, Morin *et al* 2013). Most of the world’s population lives in cities, where microclimates are influenced by the built environment, other land cover characteristics such as water, vegetation and trees, and anthropogenic processes (Duarte *et al* 2015, Wong *et al* 2016, Yang *et al* 2023). These factors contribute to phenomena such as the urban heat island (UHI), dry island, and moist island effects that make weather in cities different from the surrounding rural areas (Wang and Gong 2010, Lokoshchenko 2017, Hao *et al* 2018, Shi and Zhang 2022). Although there is a large body of work in urban microclimatology, it is mostly focused on outdoor locations (Chatzidimitriou and Yannas 2015, Piselli *et al* 2018, Jacobs *et al* 2019). To improve our understanding of urban climate and health, there is a need to contrast these findings with studies of the indoor microenvironments where people spend much of their time.

Temperature, and its effects on health, can be characterized by different metrics. Dry-bulb temperature (*T*) refers to the ambient air temperature unaffected by the moisture of the air. *T* is the standard measurement of air temperature, indicating the heat content of the air. However, extreme heat impacts human health depending on humidity (Davis *et al* 2016). In particular, high humidity limits evaporative cooling, and increases the risk of heat stress and heat-related illnesses (Mitchell *et al* 1976, Cramer *et al* 2022). Wet bulb temperature (*T*_w_), defined as the lowest temperature the air can reach through evaporative cooling, is thus an important metric for evaluating the physiological impacts of extreme heat (Justine *et al* 2023, Lu and Romps 2023). A threshold *T*_w_ of 35 °C has been proposed as the upper limit for human survivability, but other research suggests that this critical limit may be much lower under very hot and dry conditions (Vecellio *et al* 2022, Lu and Romps 2023). Therefore, it is essential to understand the spatial and temporal variability of *T* as well as *T*_w_ for assessment of human heat exposures. Previous studies have demonstrated that indoor and outdoor microclimates respond differently to surrounding built-up and landcover features (Liu *et al* 2022, Salvati *et al* 2022). In this context, distinguishing between indoor and outdoor environments is important, as they present considerably different heat exposure conditions (Quinn *et al* 2014).

In addition to direct heat impacts, variations in urban microclimates are also associated with indirect health effects such as habitat suitability for mosquitoes and transmission of mosquito-borne diseases (LaDeau *et al* 2015, Misslin *et al* 2016, Wimberly *et al* 2020). Because temperature and humidity exhibit local variations within cities, conventional weather station data cannot provide precise estimations of local exposures (Wimberly *et al* 2020, Ramsay *et al* 2021). Moreover, urban landscapes are inherently heterogeneous, encompassing variations in socio-economic conditions, vegetation cover, built density, and land use patterns as well as microclimate. Therefore, data that characterize the temporal (diurnal and seasonal) and spatial patterns of microclimate variables at the neighborhood scale are essential to assess climate-related health risks in urban neighborhoods.

Urban microclimates are influenced by land cover characteristics such as building volume, surface albedo, and vegetation cover (Bowler *et al* 2010, Kamal *et al* 2021, Smith *et al* 2023, Li *et al* 2024). Dense building volumes trap heat and limit ventilation, intensifying UHI effects, while high-albedo surfaces reflect sunlight, reducing local temperatures (Akbari *et al* 2001, Santamouris 2014). Vegetation reduces urban temperatures by providing shade, which blocks solar radiation, and through evapotranspiration, which releases water vapor to cool surrounding air (Qiu *et al* 2017, Venter *et al* 2020). Proximity to water bodies, such as rivers or lakes, moderates urban temperatures by slowly absorbing and releasing heat, creating a cold island effect and enhancing cooling through evaporation, improving thermal comfort in hot conditions (Hathway and Sharples 2012, Norton *et al* 2015).

Indoor microclimates are further influenced by the outdoor environment, but these effects are modulated by building materials, insulation, and ventilation systems (Erell and Zhou 2022). Numerous studies have explored these relationships in a wide range of urban environments (Andreou 2013, Priya and Senthil 2021, Salvati *et al* 2022, Zhang *et al* 2022). However, fewer studies have directly compared the spatial and temporal patterns of indoor and outdoor microclimates and their distinctive associations with urban land cover (Scott *et al* 2017, Asumadu-Sakyi *et al* 2019, Mukhopadhyay *et al* 2021, Salvati *et al* 2022). More research is needed to assess neighborhood-level microclimate variation at scales that are relevant to indoor and outdoor exposures and health risks in dense urban landscapes.

To address this gap, we installed a network of temperature/humidity sensors in Ahmedabad and Surat—two Indian cities—to capture fine-scale variation in temperature and atmospheric humidity. We also obtained satellite-derived estimates of building volume, surface albedo, vegetation indices, and proximity to water to understand their influence on the microclimate at varying spatial scales. The specific research questions were 1) how do indoor and outdoor air temperature (*T*) and wet bulb temperature (*T*_w_) differ in their diurnal and seasonal variability across urban neighborhoods?, 2) how are indoor and outdoor *T* and *T*_w_ associated with land cover, including building volume, vegetation, surface albedo, and water?, and 3) do indoor and outdoor *T* and *T*_w_ exhibit significantly distinct temporal and spatial patterns, indicating differential sensitivity to environmental and land cover features?

## 2. Methods

### 2.1. Study area

Our study area encompassed Ahmedabad and Surat, two major cities in Gujarat state, India. The cities have tropical climates characterized by a hot and dry season from March–June, a wet and humid monsoon season from July–September, and a cooler and drier post-monsoon season from October–February. Severe heat waves have impacted both cities in recent years, and the frequency of these events is projected to increase with climate change (Azhar *et al* 2014, Bandyopadhyay *et al* 2016, Im *et al* 2017). Despite their geographical proximity, the cities have different environmental and demographic characteristics.

Ahmedabad has a hot semi-arid climate, with average minimum and maximum temperatures of 26.3 °C and 41.4 °C during May and 13.1 °C and 28.7 °C during January. An average of three heatwave days was recorded each year during the March–May period from 2010 to 2016, including a peak temperature of 48 °C on 20 May 2016. Notably, the May 2010 heatwave resulted in a total of 1344 recorded deaths (Azhar *et al* 2014). Ahmedabad is slightly drier than Surat, with 782.2 mm of annual precipitation (WMO Climate Normals 2021). The city spans 466 sq. km and is projected to have a population of 8.16 million in 2025, with a density of 9900 persons per sq. km and an annual growth rate of 2.35% (Ahmedabad City Population | Literacy and Hindu Muslim Population 2025, Demography | Ahmedabad District, Government Of Gujarat | India 2025).

Surat, located along the Arabian Sea, has a tropical wet and dry climate, with average minimum and maximum temperatures of 26.3 °C and 36.2 °C during May and 14.7 °C and 30.9 °C during January. An average of seven heatwave days (defined as days with temperatures exceeding 40 °C) has been observed annually since 2010, with a peak of 22 such days recorded in 2010 (Magotra *et al* 2023). Total annual rainfall is approximately 1200 mm. (WMO Climate Normals 2021). Covering 462.1 sq. km, Surat has a population of 6.53 million in 2025, with a density of 10 052 persons per sq. km (2011 census) and an annual growth rate of 3.1% (Surat Municipal Corporation 2025, Surat City Population | Literacy and Hindu Muslim Population 2025).

### 2.2. Data collection and pre-processing

#### 2.2.1. Microclimate data

**Figure 1.**
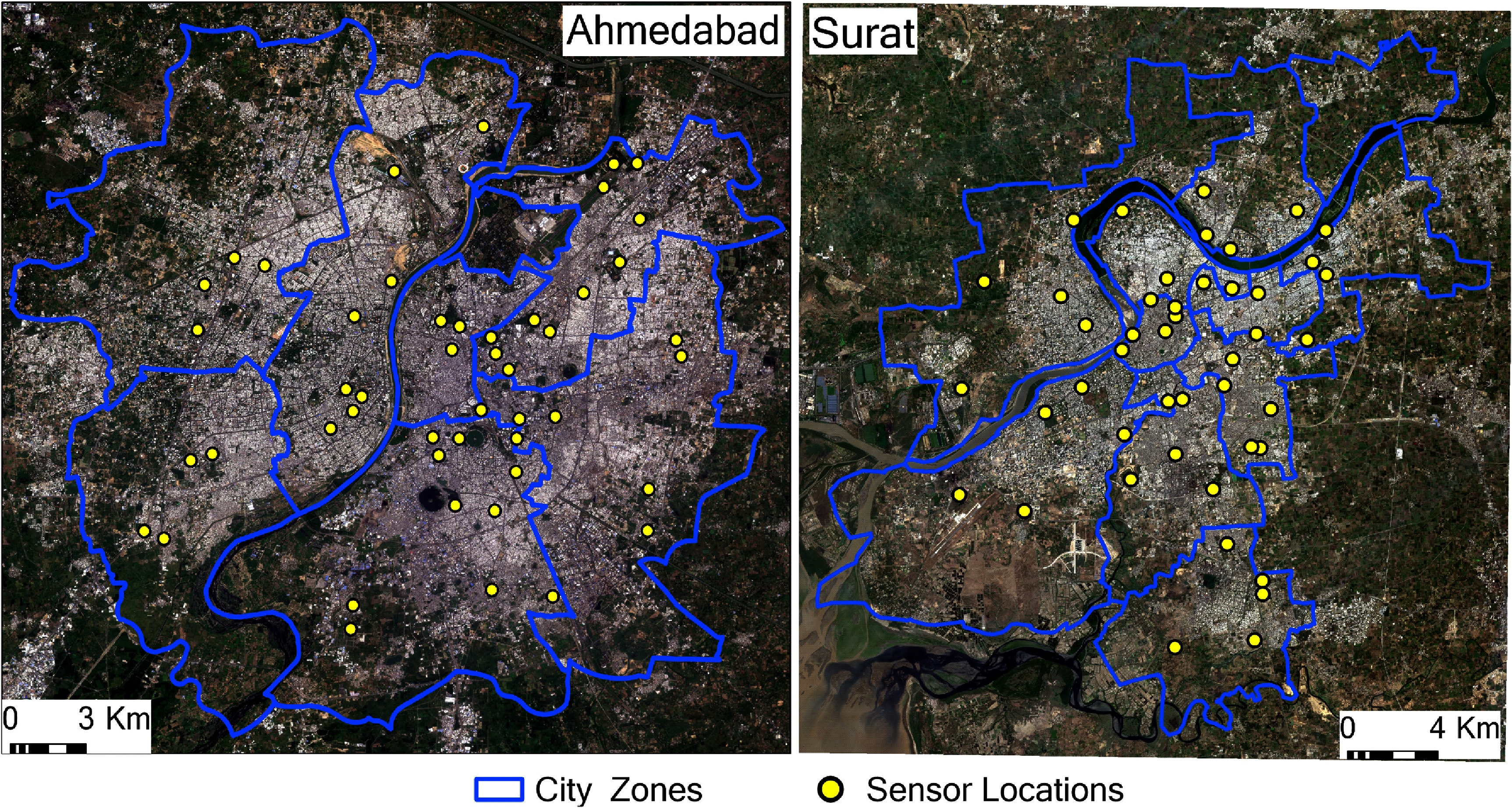
Sensor locations in Ahmedabad and Surat. There is one indoor and one outdoor sensor at each dwelling. Points are jittered to anonymize dwelling locations. Background imagery is from PlanetScope dated 5 October 2023, for Ahmedabad and 25 February 2024, for Surat (Imagery © 2024 Planet Labs Inc.).

Microclimate monitoring networks were established in both cities with Monarch Instruments Track-It temperature/humidity sensors collecting data on dry-bulb temperature (*T*) and relative humidity (*RH*) every 10 sec, which were then averaged and recorded every 30 min from 1 May 2023 through 30 April 2024. They were installed at paired indoor/outdoor locations in 48 houses in Ahmedabad and 45 houses in Surat (figure 1). These houses were involved in the vector surveillance programs in each city and were geographically stratified by administrative zone (six sensors in each of eight zones in Ahmedabad and five sensors in each of nine zones in Surat). Sensors were positioned to avoid direct sunlight and proximity to heat- or cold-emitting devices or surfaces. Indoor sensors were placed on walls of living or sleeping rooms with no air conditioners, and outdoor sensors were placed in locations near dwellings with shade from porch roofs or vegetation. Measurements were taken every half hour for a full year. Data were downloaded monthly by vector surveillance personnel using an Android mobile phone app and submitted by email for processing and analysis.

The time series of *T* and *RH* obtained from loggers were summarized as mean hourly observations. An algorithm was developed to fill data gaps (8.3% of observations in Ahmedabad and 2.2% in Surat) using data collected from other loggers during the gap periods. We imputed missing data in these gaps using time series models calibrated using data from other sensors. More details on data imputation are provided in section 1 of supplementary information. Wet bulb temperature (*T*_w_) was calculated as a function of *T* and *T*_w_ using an empirical relationship (Stull 2011).

#### 2.2.2. Satellite remote sensing data

Building volume was obtained by combining 2023 data from Google Buildings 2.5D Temporal V1-4 m rasters from Sentinel-2 imagery with building height information and Google Open Buildings V3-Polygons from very high resolution imagery (Open Buildings 2.5D Temporal Dataset 2025, Open Buildings Temporal V1 | Earth Engine Data Catalog 2025). The resulting building volume raster had a 30 m grid cell size. Harmonized Landsat and Sentinel-2 (HLS) images were obtained for Ahmedabad and Surat from May 2023 to April 2024, summarized as median monthly composites, and used to derive the normalized difference vegetation index (NDVI) (Claverie *et al* 2018). Broadband albedo was estimated from spectral bands of HLS images using Liang’s equation (Liang 2001). We computed the modified normalized difference water index (MNDWI) from HLS spectral bands and applied a threshold of 0.05 to effectively distinguish water from non-water regions (Xu 2006). Based on this threshold, we created a binary raster mask, assigning a value of 1 to pixels with MNDWI values greater than 0.05 (indicating water presence) and 0 otherwise (indicating absence of water). Procedures for building volume estimation and processing of HLS images are detailed in section 2 of the supplementary information (Jamal *et al* 2025).

### 2.3. Analysis

Seasonal and diurnal patterns of minimum and maximum *T* and *T*_w_ were assessed by graphing the time series of monthly values and summarizing the mean differences between paired indoor and outdoor loggers by month and time of day. Spatial patterns were assessed by computing the correlations between microclimate variables (minimum and maximum *T* and *T*_w_) and land cover variables (building volume, NDVI, broadband albedo, and surface water). Each variable was summarized at four focal window sizes (50 m, 100 m, 500 m, and 1000 m) around each microclimate sensor to capture effects from immediate surroundings up to neighborhood-scale influences. Monthly data for all microclimate variables and predictors were averaged seasonally, using four seasons: cold dry (January–March), hot dry (April–June), monsoon (July–September), and wet (October–December).

We calculated Spearman rank correlations to quantify the strength and direction of associations between each microclimate variable and predictor for each season and indoor versus outdoor locations. For each combination of microclimate and land cover variable, we calculated the absolute Spearman correlation for different window sizes, then averaged these values across all seasons and both indoor and outdoor locations to obtain average absolute correlation (|*ρ*|). The spatial window with the highest average absolute correlation was used to model the relationship between that specific temperature–predictor. The spatial windows with the highest |*ρ*| are explained in section 3.1 of the supplementary information.

We used generalized additive models to assess whether temporal microclimate patterns and spatial associations with land cover were distinctive at indoor versus outdoor locations. For the temporal trend models (Model 1 and Model 2), the response variables were hourly *T* and *T*_w_ and the predictor variables were a tensor product of hour of day and month. The tensor product function allowed interaction effects between the two predictor variables. In Model 2, the smoothed function differed with indoor versus outdoor location,

\begin{equation*}{\boldsymbol{Model}}1:{\text{Temp}}\sim te\left( {{\text{Hour}}\;{\text{of}}\;{\text{Day,Day}}\;{\text{of}}\;{\text{year}}} \right) + \epsilon \end{equation*}

\begin{align*} &amp; {\boldsymbol{Model}}\,2:{\text{Temp}} \nonumber\\ &amp; \quad \sim te{\text{(Hour}}\;{\text{of}}\;{\text{Day,Day}}\;{\text{of}}\;{\text{year|}}{\text{Location}}) + \epsilon .\end{align*}

For the spatial trend models (Model 3 and Model 4), the response variables were seasonal minimum and maximum *T* and *T*_w_ the predictor variables included additive smooth terms represented by thin plate regression splines for albedo, building volume, NDVI, and surface water presence, along with season as a categorical predictor. In Model 4, the smoothed functions and the seasonal variable differed with indoor versus outdoor location. The land cover variables were summarized at their respective optimal focal windows as described earlier in this section,

\begin{align*} &amp; {\boldsymbol{Model}}\,3:{\text{Temp}}. \sim s\left( {{\text{albedo}}} \right) + s\left( {{\text{buildingvolume}}} \right) \nonumber\\ &amp; \quad + s\left( {{\text{NDVI}}} \right) + s\left( {{\text{water}}} \right) + {\text{Season}} + \epsilon \end{align*}

\begin{align*} &amp; {\boldsymbol{\,Model}}\,4:{\text{Temp}}. \sim {{s}}\left( {{\text{albedo|Location}}} \right) \nonumber\\ &amp; \quad + s\left( {{\text{buildingvolume|Location}}} \right) \nonumber\\ &amp; \quad + s\left( {{\text{NDVI|Location}}} \right) \nonumber\\ &amp; \quad + s\left( {{\text{water|Location}}} \right) \nonumber\\ &amp; \quad + {\text{Season}}|{\text{Location}} + \epsilon .\end{align*}

Models were fitted separately for Ahmedabad and Surat. Because building volume and NDVI were strongly correlated, we repeated the analysis without NDVI predictor to assess whether the results were sensitive to concurvity. Model performance and fit were evaluated and compared using the adjusted coefficient of determination (adjusted *R*^2^) and the Akaike information criterion (AIC). Higher adjusted *R*^2^ values indicated better explanatory power, while lower AIC values signified improved model parsimony and fit.

## 3. Results

### 3.1. Temporal patterns of microclimate variables

**Figure 2.**
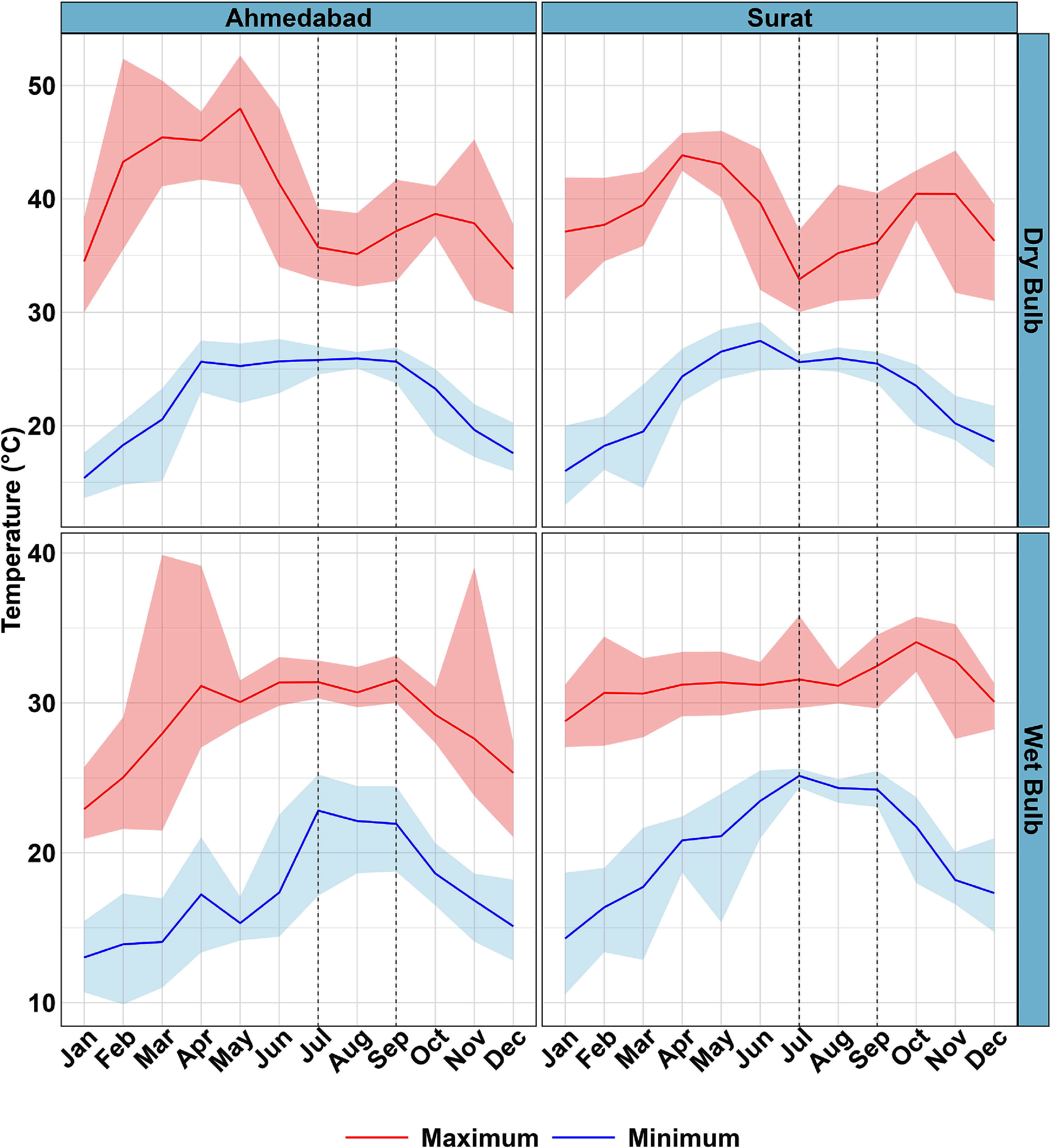
Monthly seasonality of dry and wet bulb temperatures with indoor and outdoor locations combined. May–December data are from 2023, and January–April data are from 2024. Shaded areas encompass 5%–95% quantiles. The duration between vertical dotted lines represents the monsoon season (July–September).

Marked differences were observed between the monthly variations of *T* and *T*_w_ (figure 2). While maximum *T* values were generally lowest during the monsoon, maximum *T*_w_ values tended to peak during this season. However, the behavior of maximum *T*_w_ during and around the monsoon did not vary as sharply as that of maximum *T*. The monsoon had less effect on minimum *T*, whereas minimum *T*_w_ showed sharper peaks during the monsoon.

**Figure 3.**
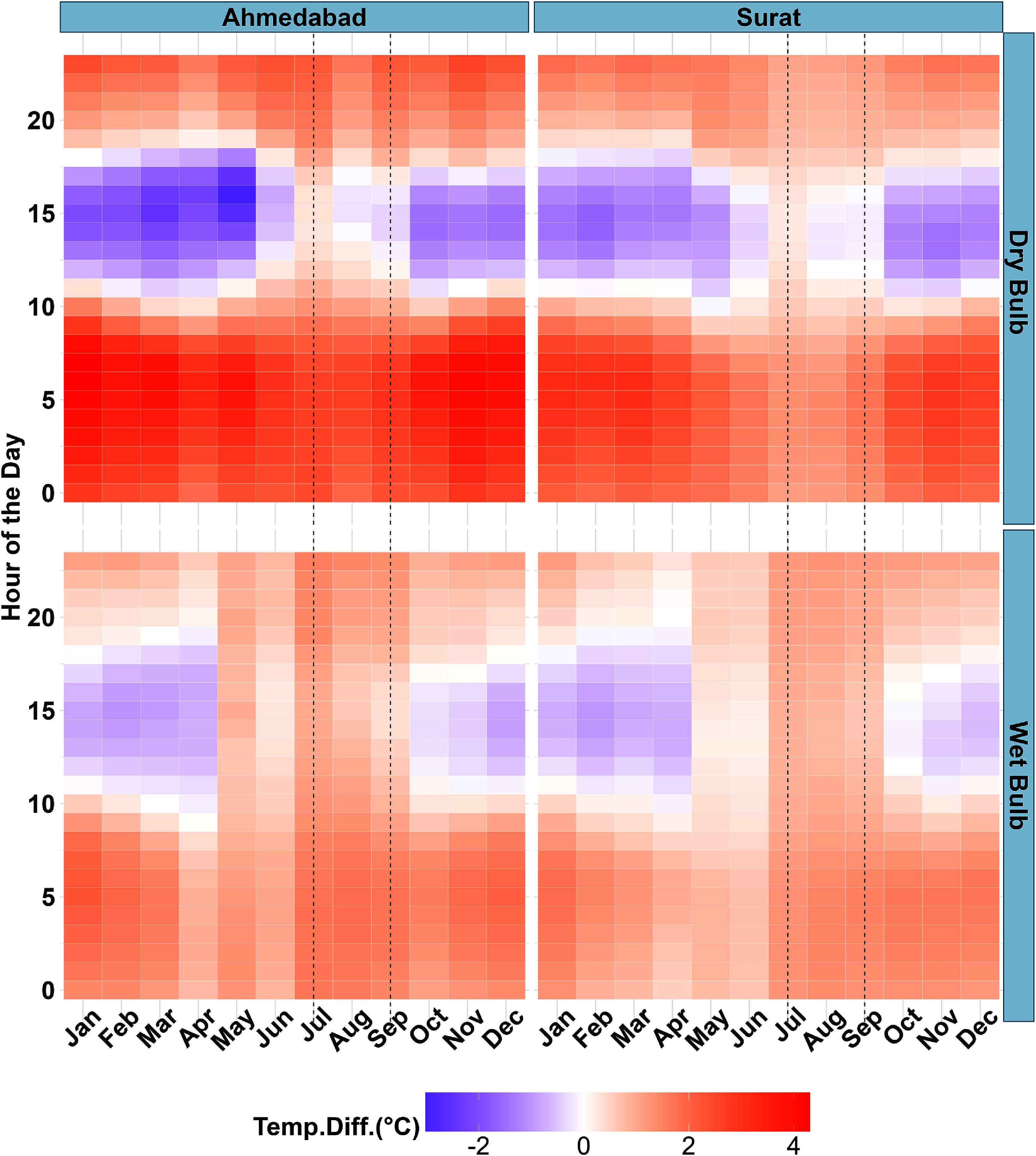
Difference between indoor and outdoor temperatures (indoor–outdoor) by month and hour of the day. May–December data are from 2023, and January–April data are from 2024. The months between vertical dotted lines represent the monsoon season (July–September).

Diurnal and monthly variations were evident when differences between paired indoor and outdoor loggers were considered (figure 3). A consistent pattern emerged in which indoor temperatures were warmer than outdoor temperatures from sunset until approximately 10 a.m. This temperature difference was less pronounced for *T*_w_ compared to *T*. This pattern reversed from around 10 a.m. to 6 p.m., when outdoor temperatures became warmer than indoor temperatures. After 6 p.m., indoor temperatures again became warmer, although the magnitude of this difference decreased compared to morning. However, this reversal pattern was not as evident during the monsoon season, as the differences between indoor and outdoor temperatures became minimal between noon (12 p.m.) and 6 p.m. The reversal effect was not observed for *T*_w_ during the monsoon.

### 3.2. Correlations between microclimate variables and landscape metrics

**Figure 4.**
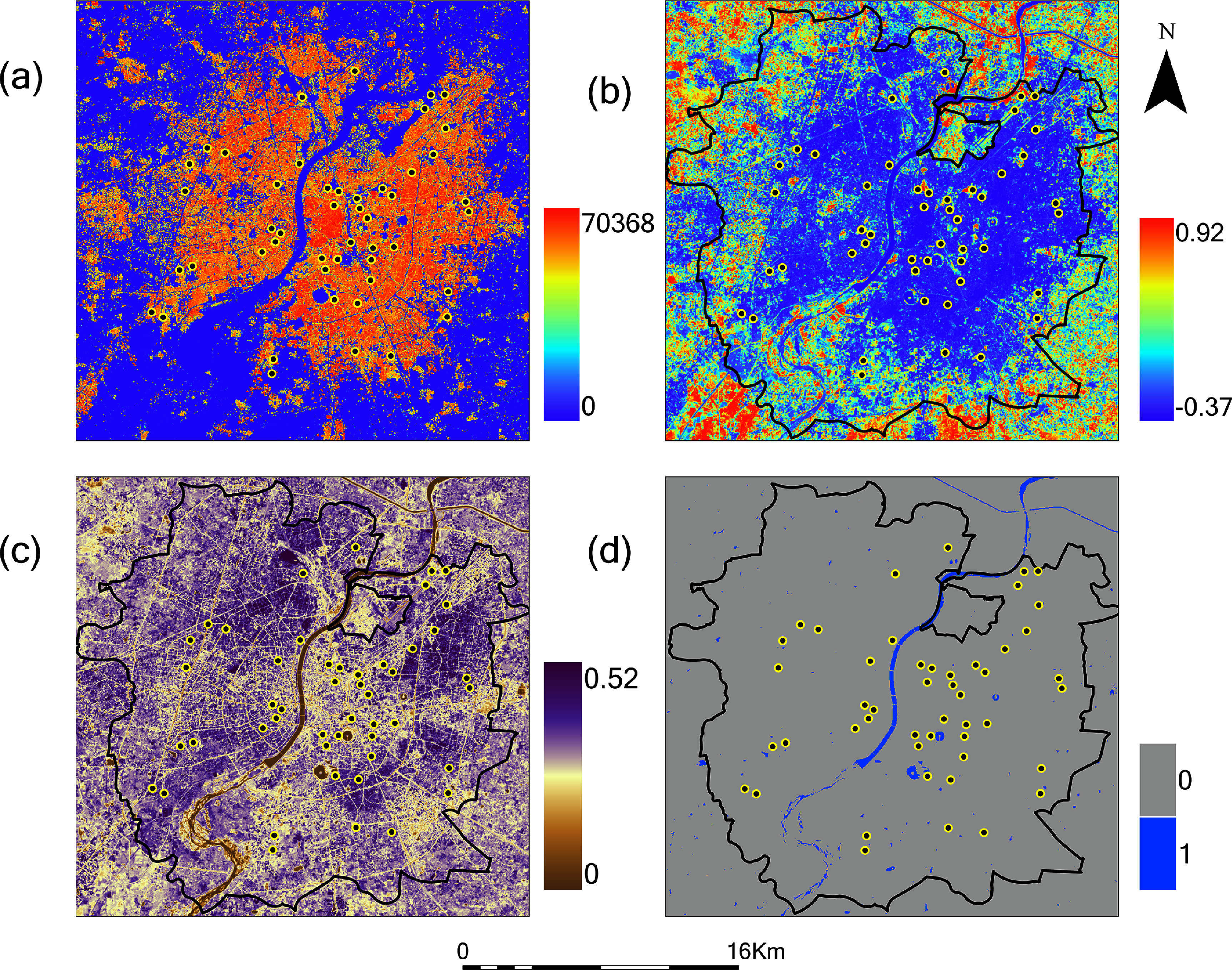
Satellite-derived measurements of land cover for Ahmedabad-(a) building volume (in m^3^ per 30 m grid cell), (b) vegetation (NDVI) for October 2023, (c) surface reflectivity (albedo) for October 2023, and (d) water presence for October 2023 (MNDWI for water presence,1 indicates MNDWI > 0.05, 0 indicates MNDWI < 0.05). Black line represents Ahmedabad city boundary. Black dots with yellow outline represent sensor locations inside Ahmedabad city.

**Figure 5.**
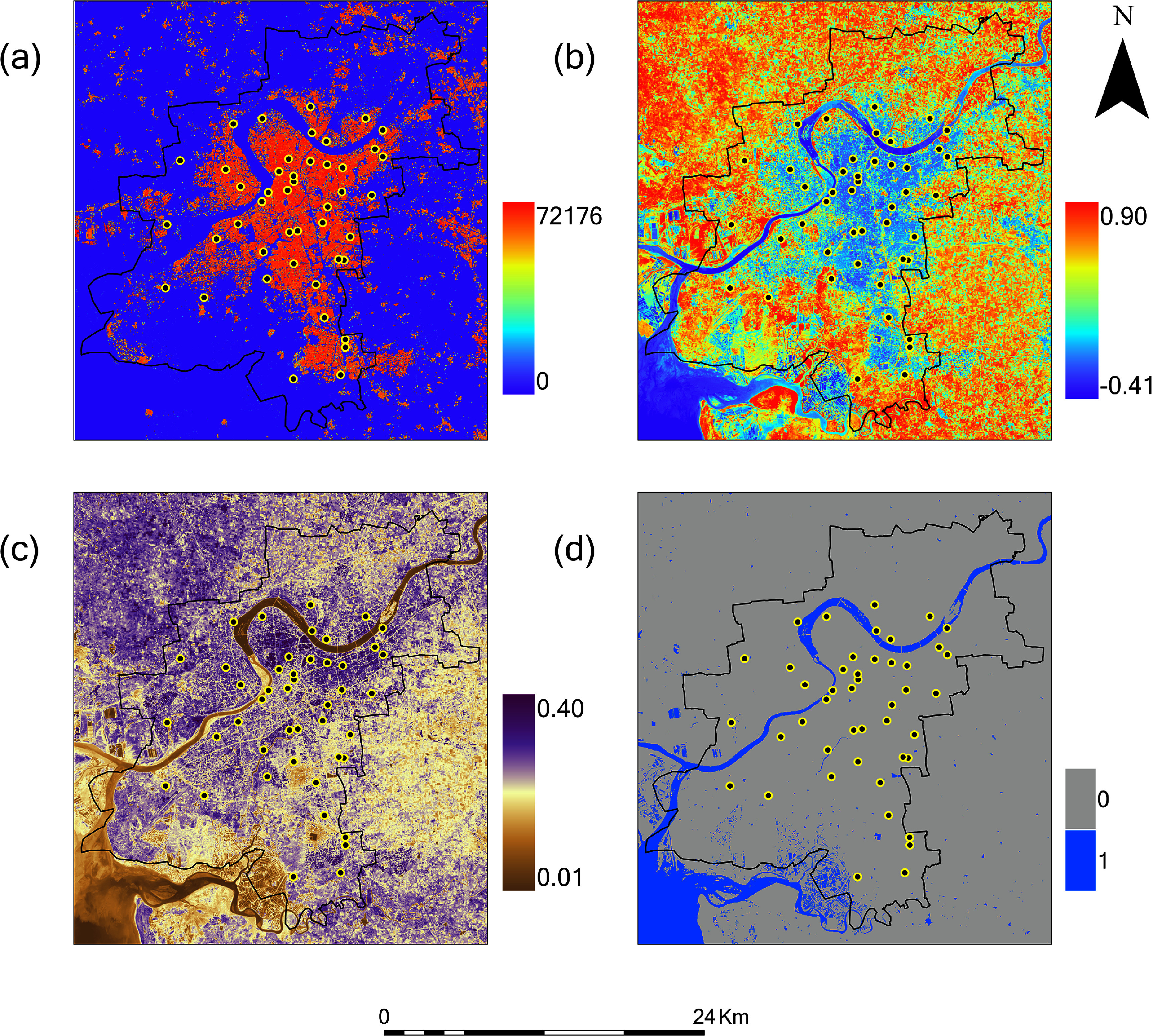
Satellite-derived measurements of land cover for Surat-(a) building volume (in m^3^ per 30 m grid cell), (b) vegetation (NDVI) for October 2023, (c) surface reflectivity (albedo) for October 2023, and (d) water presence for October 2023 (MNDWI for water presence,1 indicates MNDWI > 0.05, 0 indicates MNDWI < 0.05). Black line represents Surat city boundary. Black dots with yellow outline represent sensor locations inside Surat city.

There was decreasing building volume and increasing NDVI along a gradient from the city center to the periphery in both Ahmedabad (figure 4) and Surat (figure 5). As a result, building volume and NDVI at the sensor locations was strongly correlated (Spearman correlation −0.80 to −0.83 for Ahmedabad, and −0.78 to −0.80 for Surat when both variables were summarized with a focal window of 100 m). Patterns of albedo captured finer-scale variation in the reflective properties of buildings, vegetation and soil. Water reflected the central river bisecting each city as well as smaller ponds for water storage, recreation, and aquaculture.

The focal window size with the highest mean absolute correlation was determined for each combination of city, land cover variable, and temperature variable (*T* or *T*_w_). The correlations for these windows are shown in figure 6 for Ahmedabad and figure 7 for Surat. Correlations for all window sizes and optimal window sizes are shown in section 3 of the supplementary information (figures S3–S11).

**Figure 6.**
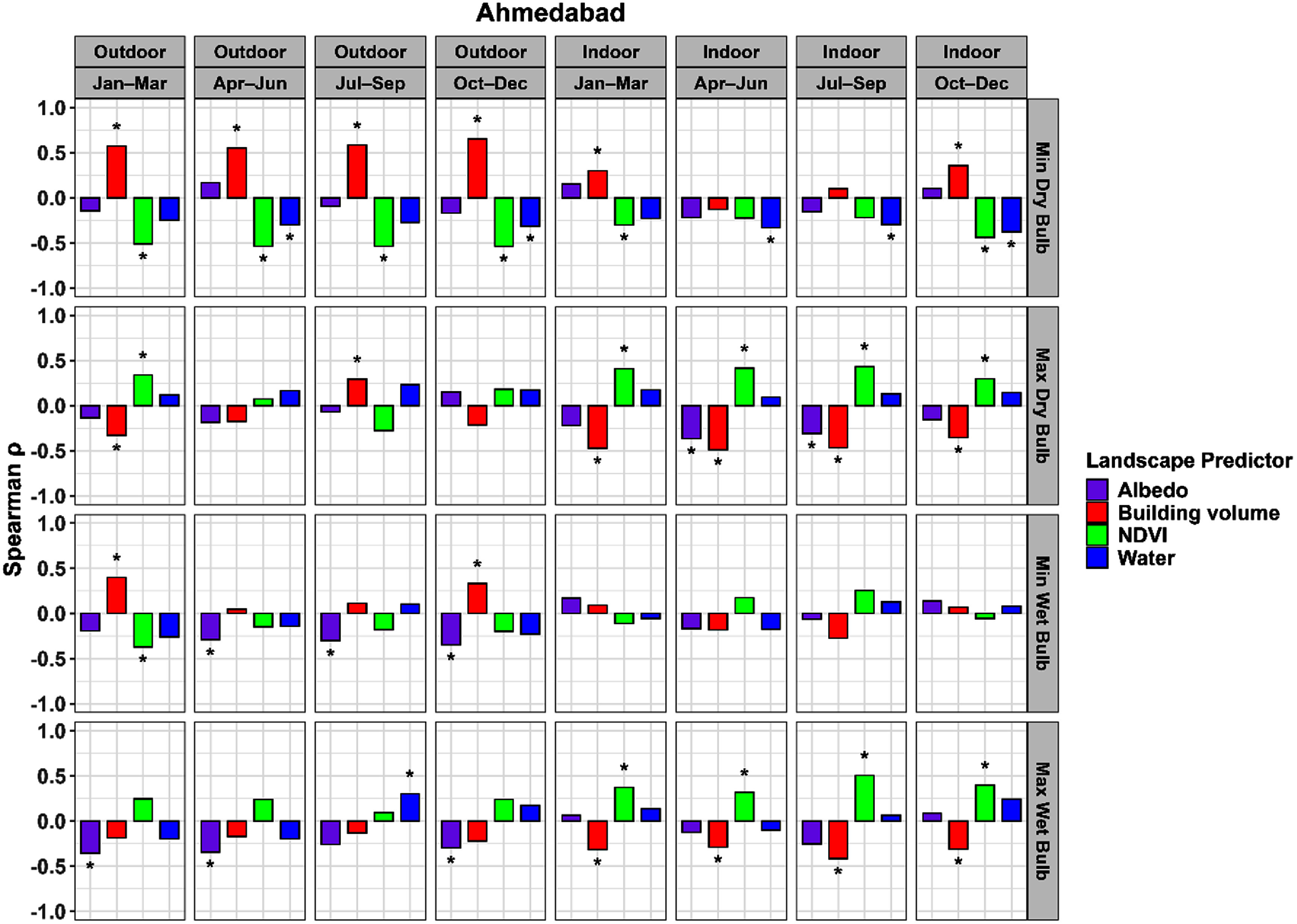
Spearman correlations between microclimate variables and predictors for Ahmedabad. The stars above (or below) the bars show statistical significance at an alpha level of 0.05.

**Figure 7.**
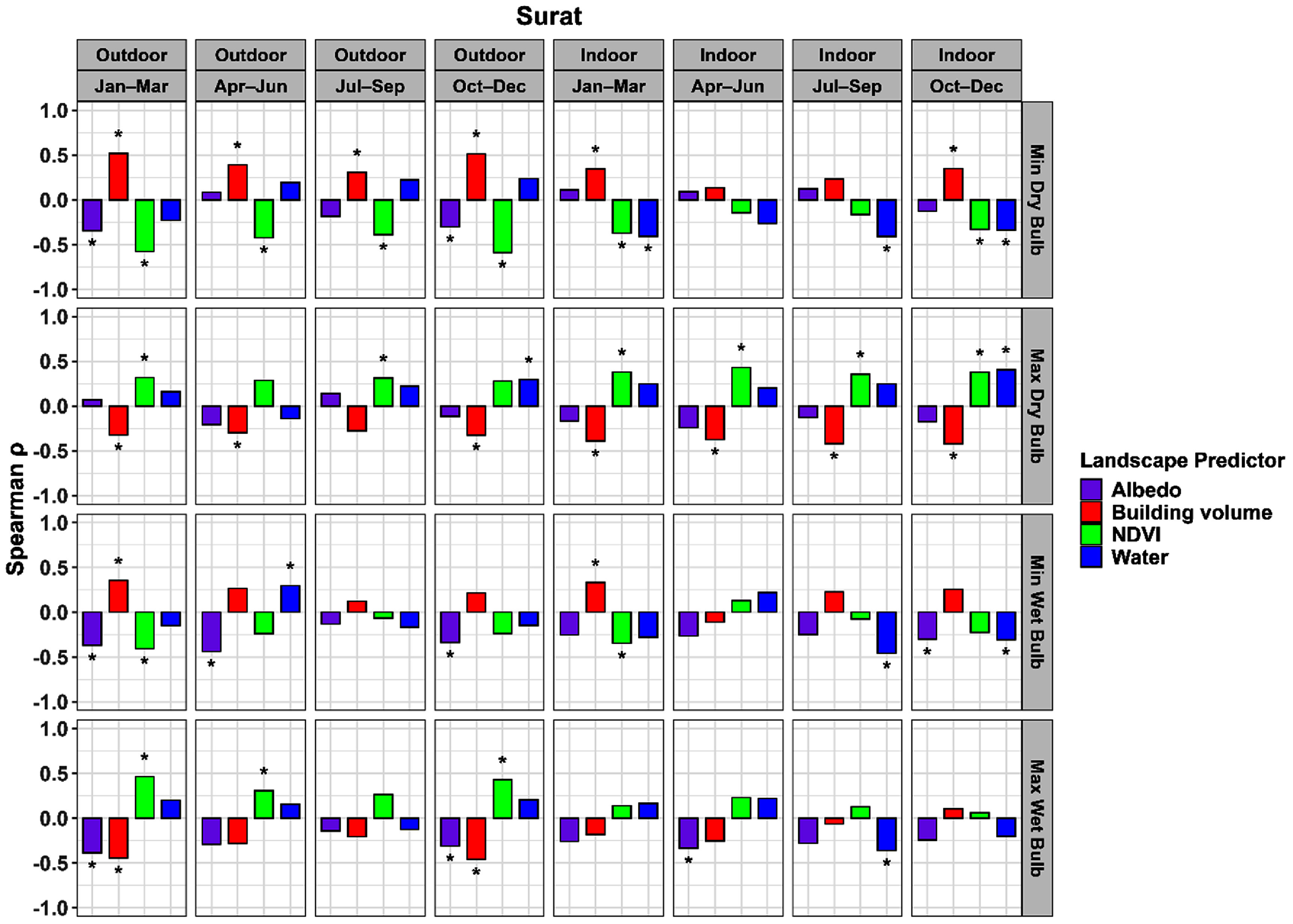
Spearman correlations between microclimate variables and predictors for Surat. The stars above (or below) the bars show statistical significance at an alpha level of 0.05.

Among the four land cover variables, building volume showed the most consistent correlation patterns across both Ahmedabad (figure 6) and Surat (figure 7). Correlations were generally stronger in outdoor settings than indoors, especially for minimum *T* and *T*_w_. Outdoor minimum *T* had the highest positive correlations across all seasons, while indoor minimum *T* also displayed significant but lower positive correlations, particularly during January–March and October–December. In contrast, maximum *T* and *T*_w_ tended to have negative associations with building volumes. Overall, correlations were stronger and more significant for *T* than *T*_w_.

Compared to building volume, NDVI exhibited inverse patterns of association with *T* and *T*_w_ and across both cities. Correlations were generally stronger in outdoor environments, particularly for minimum *T* and *T*_w_, where NDVI showed consistent negative relationships across seasons. The highest outdoor correlations were observed with minimum *T and T*_w_. Indoor correlations were lower with less seasonal consistency. For maximum *T* and *T*_w_, NDVI exhibited significant positive associations both indoors and outdoors.

It is notable that indoor maximum *T* was negatively correlated with building volume and positively correlated with NDVI, a pattern that contrasted with the expected cooling effect of vegetation. The observed effects were consistent across both cities for indoor temperature, and similar associations were also observed outdoors, although with fewer significant relationships. In Ahmedabad, maximum *T*_w_ also exhibited stronger correlations indoors, but weaker and insignificant correlations outdoors. In Surat, maximum *T*_w_ exhibited stronger correlations outdoors compared to indoors.

Albedo showed consistently negative relationships with *T* and *T*_w_. These associations displayed less variation across indoor and outdoor settings compared to building volume and NDVI. Notably, outdoor *T*_w_ had higher correlations with albedo than outdoor *T*, particularly in Ahmedabad.

Water presence, represented by masked MNDWI, had some significant correlations at the largest spatial window of 1000 m. Consistent negative correlations were observed for indoor and outdoor minimum *T* across both cities. Correlations with other temperature variables were generally lower and more variable. In Surat, water exhibited stronger and more statistically significant correlations with *T*_w_ than Ahmedabad.

### 3.3. Modeling the influence of location in the spatial and temporal variation of microclimate variables

#### 3.3.1. Modeling temporal variation

The models explained substantial portions of the temperature variation (table 1). In both cities, higher adjusted *R*^2^ values and lower AIC values were observed for Model 2 for *T* and *T*_w_, indicating distinctive patterns of diurnal and seasonal variation at indoor versus outdoor locations. The adjusted *R*^2^ values were all slightly higher for Surat than Ahmedabad, indicating that the diurnal and season trends were stronger and explained more of the variation in *T* and *T*_w_.

**Table 1.** Model fit statistics for GAMs showing temporal variation in dry-bulb temperature (*T*) and wet-bulb temperature (*T*_w_), where Model 1 does not include location information and Model 2 distinguishes between indoor and outdoor locations. Fit statistics include adjusted *R*^2^ and Akaike’s information criterion (AIC). Change in AIC shows AIC_Model 2_–AIC_Model 1_.

Temperature variable		Model 1		Model 2		Change in AIC
	Location	*R* ^2^	AIC	*R* ^2^	AIC	
*T*	Surat	0.70	3817 968	0.74	3696 508	−121 460
*T*	Ahmedabad	0.65	3488 610	0.68	3422 873	−65 737
*T*_w_	Surat	0.78	3482 199	0.79	3447 554	−34 645
*T*_w_	Ahmedabad	0.69	3307 251	0.70	3283 705	−23 546

#### 3.3.2. Modeling spatial variation

In both Ahmedabad (table 2) and Surat (table 3), Model 4 consistently outperformed Model 3 across all temperature metrics (minimum and maximum *T* and *T*_w_). Model 4 achieved consistently higher *R*^2^ values and lower AIC values, indicating better model fit when land cover associations were allowed to vary across indoor versus outdoor locations. The differences were most pronounced for minimum *T* and *T*_w_ in both cities, with substantial AIC reductions. All differences between Model 3 and Model 4 were statistically significant (ANOVA, *p* < 0.05). Similar results were obtained after fitting models 3A and 4A, which excluded NDVI as a predictor (tables 2(A) and 3(A) in section 4 of the supplementary information, demonstrating that the correlation between NDVI and building volume did not bias our conclusions.

**Table 2.** Model fit statistics for GAMs showing spatial variation for dry bulb temperature (*T*), where Model 3 does not include location information and Model 4 distinguishes between indoor and outdoor locations. Fit statistics include adjusted *R*^2^ and Akaike’s information criterion (AIC). Change in AIC shows AIC_Model 4_–AIC_Model 3_.

Temperature metric	Location	Model 3		Model 4		Change in AIC
		*R* ^2^	AIC	*R* ^2^	AIC	
Minimum *T*	Ahmedabad	0.66	1619.25	0.94	988.23	−631.02
Maximum *T*	Ahmedabad	0.71	1522.43	0.79	1417.65	−104.78
Minimum *T*	Surat	0.72	1332.55	0.89	1014.73	−317.82
Maximum *T*	Surat	0.59	1471.04	0.63	1449.57	−21.47

**Table 3.** Model fit statistics for GAMs showing spatial variation for wet bulb temperature (*T*_w_), where Model 3 does not include location information and Model 4 distinguishes between indoor and outdoor locations. Fit statistics include adjusted *R*^2^ and Akaike’s information criterion (AIC). Change in AIC shows AIC_Model 4_–AIC_Model3_.

Temperature Metric	Location	Model 3		Model 4		Change in AIC
		*R* ^2^	AIC	*R* ^2^	AIC	
Minimum *T*_w_	Ahmedabad	0.94	1042.04	0.97	769.52	−272.52
Maximum *T*_w_	Ahmedabad	0.94	898.85	0.95	850.29	−48.56
Minimum *T*_w_	Surat	0.93	897.72	0.96	731.00	−166.72
Maximum *T*_w_	Surat	0.83	1030.56	0.86	979.15	−51.41

## 4. Discussion

Our findings show that building volume was positively correlated with minimum nighttime *T*, with stronger relationships outdoors, highlighting an UHI effect driven by heat retention in densely built areas (Santamouris 2014, Hsu *et al* 2021). Vegetation, quantified via NDVI, was negatively associated with nighttime *T*, particularly outdoors, as areas with high NDVI typically correspond to lower building volume and reduced impervious surface coverage, allowing them to cool more efficiently at night due to lower thermal inertia (Bowler *et al* 2010, Meili *et al* 2021). Weaker indoor correlations with both building volume and NDVI suggest that the insulating effects of structures moderate temperature extremes (Qiu *et al* 2017, Salvati *et al* 2022). These results generally agree with previous studies in a variety of cities that have found that measures of urbanization density are typically associated with higher minimum dry-bulb temperature (Oleson *et al* 2015, Chapman *et al* 2017, Logan *et al* 2020). Correlations between building volume and minimum *T*_w_ had similar patterns but were weaker, indicating that moist heat stress, is sensitive to other factors that constrain the patterns.

Maximum *T* was also associated with building volume and NDVI. In contrast to minimum *T*, the correlations were reversed, so that maximum *T* at both indoor and outdoor locations was warmest in areas with low building volume and high NDVI. Because of the underlying negative correlation between building density and NDVI, this effect reflected an urban to rural gradient with lowest maximum *T* in the densely developed urban core and the highest maximum *T* at more isolated dwellings near the urban periphery. These findings contrast with the general expectation that daytime temperature, including land surface temperature as well as air temperature, will be lowest in vegetated areas because of cooling effects of evapotranspiration and shade (Mildrexler *et al* 2011, Napoli *et al* 2016, Qiu *et al* 2017). As with minimum temperature, these patterns are generally consistent between *T* and *T*_w_, although there are fewer statistically significant correlations with *T*_w_.

Previous studies have demonstrated that urban morphology has a significant influence on indoor thermal environments (Robinson 2006, Mirzaei *et al* 2012). A modeling study conducted in urban Singapore concluded that increased building density can reduce indoor temperatures during daytime, primarily due to the shielding effect, whereby closely spaced buildings block direct solar radiation and thus reduce indoor heat gain (Li *et al* 2022). Furthermore, the influence of building density on indoor air temperatures tends to be more pronounced during the day than at night, when solar exposure is minimal (Mills 1997). On the other hand, locations characterized by lower building density and higher NDVI generally have a higher sky view factor, which has been linked to elevated indoor temperatures during daytime due to greater exposure to direct solar radiation (Zheng and Li 2022).

The results for maximum temperature should be interpreted in the context of our microclimate sampling design. Sensors were located at individual dwellings, which ranged from ground-floor apartments in the urban core to blocks of detached houses in outlying neighborhoods to more isolated dwellings at the urban fringe. Outdoor sensors were placed on or near and exterior wall of a house, typically in a shaded area on a porch. Thus, the outdoor temperatures represent microclimates in close proximity to buildings and may be sensitive to similar insulating effects as indoor temperatures during the day. Also, vegetation cover within these cities was generally low, and our sampling design did not incorporate locations close to parks or other places with urban vegetation densities. Thus, our finding of higher temperatures in locations with higher NDVI does not mean that vegetation cooling is not occurring in other areas, but it does suggest that cooling effects related to building density are more important in some indoor locations and outdoor spaces proximal to those buildings.

Surface albedo exhibited significant negative correlations with temperature, most commonly for *T*_w_ and at outdoor locations, indicating that more reflective buildings tended to have lower temperatures. High-albedo materials reflect a greater portion of incoming solar radiation, thereby reducing heat absorption at the surface. This negative association supports the concept of cool roofs, designed to mitigate urban heat (Sharma *et al* 2016, Wang *et al* 2022). However, these albedo effects are contingent on a variety of other factors including urban canyon effects, multiple reflections and shadowing (Akbari *et al* 2001, Chatzidimitriou and Yannas 2015). There were some negative correlations observed between minimum *T* and water presence at 1000 m spatial scale and these likely reflect the cold island effect produced by rivers (He *et al* 2024). Additionally, the urban morphology surrounding rivers and the configuration of adjacent green spaces can influence the strength of the cold island effect, potentially explaining the differing behaviors observed between Ahmedabad and Surat (Saaroni and Ziv 2003, Wang and Chen 2025).

*T*_w_ showed different associations with landscape predictors than *T* because *T*_w_ depends on atmospheric humidity in addition to air temperature. The effects of land cover on humidity are different from effects on temperature, reflecting local inputs sources from evapotranspiration combined with dynamic mixing of air (Findell *et al* 2017, Rahman *et al* 2022, Zhang *et al* 2023, Chagnaud *et al* 2025). For example, the negative correlations that we observed between albedo and *T*_w_ may reflect reduced evapotranspiration and humidity in areas with dense buildings and concrete surfaces in addition to reflectance of incoming solar radiation. Because of the combined physical mechanisms through which surface energy and moisture exchange processes affect temperature and humidity, land cover relationships are complex and represent an important area for further research.

Many previous studies comparing indoor and outdoor urban microenvironments lacked a spatially representative distribution of sensors across the study area, such as sensor placements that were clustered in specific parts of the city rather than being spread across the urban-to-rural gradient. In addition, many studies only collected data for short periods, ranging from a few days or a month, which is not enough to capture seasonal microclimate patterns (Nguyen and Dockery 2016, Moghbel and Erfanian Salim 2017, Tasgaonkar *et al* 2022). In contrast, our study stratified sensors across zones within each city and covers a full year of data collection, enabling detailed exploration of spatiotemporal trends. Moreover, our methodology of pairing indoor and outdoor sensors provides precise estimations of the differences in microenvironmental conditions at each dwelling.

Our study expands upon previous work focused on *T* by additionally considering diurnal and spatial variations in *T*_w_, thus demonstrating broader insights. Compared to *T*, fewer studies have studied spatiotemporal variation in *T*_w_, specifically in the context of assessing indoor heat stress (Gaspar and Quintela 2009, Hall *et al* 2022). Previous interpretations of *T*_w_ have focused on threshold values of human thermal comfort (Epstein and Moran 2006, Abdel-Ghany *et al* 2013, Vecellio *et al* 2022). In Ahmedabad and Surat, *T*_w_ exhibits stronger diurnal and seasonal variation patterns than *T*, and land cover associations with *T*_w_ are distinctive from those with *T*. These observations have implications in tropical regions where air conditioning is uncommon (Amaripadath *et al* 2023).

Without adequately accounting for humidity, the extent and magnitude of heat stress exposure, especially in informal settlements, could be significantly underestimated (Mukhopadhyay *et al* 2021). Understanding indoor humidity patterns is particularly important because *T*_w_ directly affects the body’s ability to cool through sweating, and high *T*_w_ indoors can increase heat stress for vulnerable groups who spend most of their time inside (Vecellio *et al* 2022). During the pre-monsoon season, high temperatures combined with poor ventilation can create dangerously warm indoor conditions at night, limiting recovery from daytime heat (Sampson *et al* 2013, Hampo *et al* 2024). In the monsoon season, even when outdoor temperatures fall, persistently high *T*_w_ indoors reduces evaporative cooling, intensifying health risks (Ambika *et al* 2024, Masuda *et al* 2024).

This study focused on contrasting temperature in indoor versus outdoor environments in the context of diurnal and season trends and land cover effects on spatial patterns. Because remotely sensed land cover data are spatially contiguous and widely accessible, they are important for understanding and predicting the spatial patterns of urban heat (Rotem-Mindali *et al* 2015, Coutts *et al* 2016, Bonafoni *et al* 2017). Our results emphasize that indoor and outdoor temperatures have different land cover associations, and additional sources of information will be needed to better understand and predict these indoor patterns. Adaptive human behaviors such as the use of fans, window shutters, or sleeping positions all have the potential to mitigate indoor heat. Additionally, local data on building materials, and socioeconomic characteristics can help to explain more of the spatial variability in urban microclimates. Collection of these additional data sources can support more nuanced understanding of the proximal drivers influencing spatial variations in urban microclimate and provide a foundation for high-resolution predictive modeling of urban microclimate (Buyantuyev and Wu 2010, Wong *et al* 2016, Deng *et al* 2024).

## 5. Conclusions

Our findings demonstrate that it is important to distinguish between indoor and outdoor locations when studying the effects of urban land cover on dry and wet-bulb temperatures. In particular, we found that maximum indoor temperatures were coolest in neighborhoods with high building volume and low vegetation cover, running counter to the general expectation that urban vegetation will have a cooling effect. These insights are important for future efforts at predictive modeling of microclimate based on land cover and can inform urban planning and design strategies to mitigate heat and humidity, improving public health outcomes in tropical cities.

## Data Availability

The data that support the findings of this study are openly available at the following URL/DOI: https://doi.org/10.6084/m9.figshare.29236661. Supplementary Methods and Results available at https://doi.org/10.1088/1748-9326/ae09bc/data1.
